# Ants as ecological indicators of rainforest restoration: Community convergence and the development of an Ant Forest Indicator Index in the Australian wet tropics

**DOI:** 10.1002/ece3.2992

**Published:** 2017-09-12

**Authors:** Michael J. Lawes, Anthony M. Moore, Alan N. Andersen, Noel D. Preece, Donald C. Franklin

**Affiliations:** ^1^ Research Institute for the Environment and Livelihoods Charles Darwin University Darwin NT Australia; ^2^ Department of Geography McGill University Montreal QC Canada; ^3^ CSIRO Land & Water Flagship Darwin NT Australia; ^4^ Centre for Tropical Environmental & Sustainability Science College of Science & Engineering James Cook University Townsville Qld Australia; ^5^ Biome5 Pty Ltd. Atherton Qld Australia

**Keywords:** functional groups, habitat condition, indicator species, rainforest restoration, succession

## Abstract

Ecosystem restoration can help reverse biodiversity loss, but whether faunal communities of forests undergoing restoration converge with those of primary forest over time remains contentious. There is a need to develop faunal indicators of restoration success that more comprehensively reflect changes in biodiversity and ecosystem function. Ants are an ecologically dominant faunal group and are widely advocated as ecological indicators. We examine ant species and functional group responses on a chronosequence of rainforest restoration in northern Australia, and develop a novel method for selecting and using indicator species. Four sampling techniques were used to survey ants at 48 sites, from grassland, through various ages (1–24 years) of restoration plantings, to mature forest. From principal components analysis of seven vegetation metrics, we derived a Forest Development Index (FDI) of vegetation change along the chronosequence. A novel Ant Forest Indicator Index (AFII), based on the occurrences of ten key indicator species associated with either grassland or mature forest, was used to assess ant community change with forest restoration. Grasslands and mature forests supported compositionally distinct ant communities at both species and functional levels. The AFII was strongly correlated with forest development (FDI). At forest restoration sites older than 5–10 years that had a relatively closed canopy, ant communities converged on those of mature rainforest, indicating a promising restoration trajectory for fauna as well as plants. Our findings reinforce the utility of ants as ecological indicators and emphasize the importance of restoration methods that achieve rapid closed‐canopy conditions. The novel AFII assessed restoration status from diverse and patchily distributed species, closely tracking ant community succession using comprehensive species‐level data. It has wide applicability for assessing forest restoration in a way that is relatively independent of sampling methodology and intensity, and without a need for new comparative data from reference sites.

## 1 Introduction

Ecosystem restoration plays an increasingly important role in the global response to widespread deforestation and land degradation (Chazdon, 2008; Food and Agriculture Organization of the United Nations 2010). In recent decades, clearing of tropical forests has proceeded at an alarming rate (Hansen et al., 2013; Millennium Ecosystem Assessment 2005; Sloan, Jenkins, Joppa, Gaveau, & Laurance, 2014), prompting international interest in tropical forest restoration (Aide, Zimmerman, Pascarella, Rivera, & Marcano‐Vega, 2000; Ashton, Gunatilleke, Singhakumara, & Gunatilleke, 2001; Chapman & Chapman, 1999; Leopold, Andrus, Finkeldey, & Knowles, 2001). There is widespread debate about the extent to which regenerating and reforested habitats can sustain tropical biodiversity (Ashton et al., 2001; Ruiz‐Jaen & Aide, 2005; Wright & Muller‐Landau, 2006).

To benefit forest biodiversity, restoration must develop along an ecological pathway that converges with natural forest (Le, Smith, Herbohn, & Harrison, 2012; Reay & Norton, 1999). An underlying assumption in ecological restoration is that forest fauna will recolonize as vegetation becomes established, and that ecosystem function and forest biodiversity will thereby converge on the mature forest condition (Kanowski, Catterall, Freebody, Freeman, & Harrison, 2010; Reay & Norton, 1999). However, the extent to which faunal communities of forests undergoing restoration converge with those of primary forest over time is highly contentious (Moir, Brennan, Koch, Majer, & Fletcher, 2005). The responses of forest fauna to the habitat changes associated with restoration are varied and complex (Gibb & Cunningham, 2009; Nakamura, Proctor, & Catterall, 2003; Whitehead, Goosem, & Preece, 2014), and vegetation structure is often a poor surrogate of faunal communities even in natural systems (Brown & Williams 2016). Although habitat structure provides the necessary framework for faunal recolonization (Smith et al., 2008), measurements of vegetation alone can provide misleading assessments of restoration success. To ensure that restoration can be designed and managed for successful biodiversity outcomes, it is important to incorporate fauna into metrics for assessing and predicting restoration trajectories (González, Rochefort, Boudreau, & Poulin, 2014; McAlpine et al., 2016).

It is widely recognized that species composition provides a more robust measure of restoration success than do simple community metrics such as species richness (Andersen & Majer, 2004; Reid, 2015; Solar et al., 2016). However, the use of species‐level information is often also problematic, because species responses to restoration may vary widely among taxa (Holt & Miller, 2010; Laurance, 1994; Smith et al., 2008; Young et al., 2013), and highly diverse communities often have naturally high‐species turnover (Giller, 1996; Suganuma & Durigan, 2015). An alternative approach is to base assessments on functional rather than species composition (Andersen, 1990; Brancalion & Holl, 2016), but such an approach can be overly coarse if the restoration goal is to re‐establish the full complement of species.

Invertebrates are often used as indicators of ecological change in terrestrial ecosystems because of their critical roles in ecosystem function and dominant contribution to faunal diversity (Brown, 1997; Kremen, 1992; Lawes, Kotze, Bourquin, & Morris, 2005; McGeoch, 1998; Uehara‐Prado et al., 2009). In particular, ants have been widely promoted as bioindicators because they are highly abundant, easily sampled, closely connected with ecosystem function, and their responses to habitat disturbance are better understood than those of most other invertebrate groups (Andersen, 1999; Andersen & Majer, 2004; Folgarait, 1998). This includes a well‐developed understanding of functional change in ant communities, based on functional groups that respond predictably to environmental stress and disturbance (Andersen, 1995; Andersen & Majer, 2004; Hoffmann & Andersen, 2003). Ant communities have been extensively used to assess a range of restored habitat types, with changes in ant species and functional composition consistently indicating the successional stage and ecological condition of restoration (Andersen, Hoffmann, & Somes, 2003; Andersen & Majer, 2004). Ant community composition can thus inform whether the trajectory of restoration is converging on mature ecosystems or following alternative pathways.

Here, we examine ant species and functional group responses to rainforest restoration in the World Heritage‐listed Australian Wet Tropics (AWT). There has been considerable interest in understanding ant responses to land clearing and reforestation in the region (Catterall et al., 2004; King, Andersen, & Cutter, 1998; Leach et al., 2013; Piper, Catterall, Kanowski, & Proctor, 2009). However, we do not have a predictive understanding of ant successional dynamics in relation to forest restoration and have not identified robust ant indicators that can be applied broadly in the assessment of restoration success. Our study uses a chronosequence (space‐for‐time substitution) to address three key objectives. First, we develop a Forest Development Index (FDI) that quantifies vegetation change along the chronosequence, as a basis for assessing ant community change in relation to vegetation restoration. Second, we document the extent to which ant species and functional composition at sites undergoing restoration have converged on that of mature rainforest. Third, we develop a novel method for selecting and using indicator species that allows for the assessment of the successional status of other sites undergoing restoration in the region, largely independently of differences in sampling methodology and without a need for further sampling of reference sites.

## 2 Methods

### 2.1 Study area and sites

The study was undertaken on the Atherton Tablelands, North Queensland, (17°14′–17°27′S; 145°30′–145°40′E) in the Australian Wet Tropics region. The native vegetation was tropical mid‐elevation rainforest; much has been cleared and small patches of rainforest and reforestation now exist in a matrix of pasture‐dominated agriculture. Large tracts of mature rainforest remain adjacent to the study region. Annual rainfall varies from 1,300 to 3,000 mm across the Tablelands on a decreasing SE‐NW gradient. Rainfall occurs year round but is highest in the summer.

Ant communities in AWT rainforests have distinctive species composition that contrasts with that in adjacent open sclerophyll habitats (van Ingen, Campos, & Andersen, 2008). The thermophilic species of *Iridomyrmex* (functional group: Dominant Dolichoderinae) that dominate ant communities of open habitats throughout Australia are absent from rainforest habitats. The most common epigaeic ants belong to the functional groups Generalized Myrmicinae (esp. *Pheidole* spp.) and Opportunists (esp. *Rhytidoponera* spp. and *Nylanderia* spp.). Habitat clearing for pasture favors Opportunists, promotes colonization by *Iridomyrmex*, and eliminates most species from the functional groups Tropical‐Climate Specialists and Specialist Predators (Andersen, 2000; King et al., 1998).

**Figure 1:**
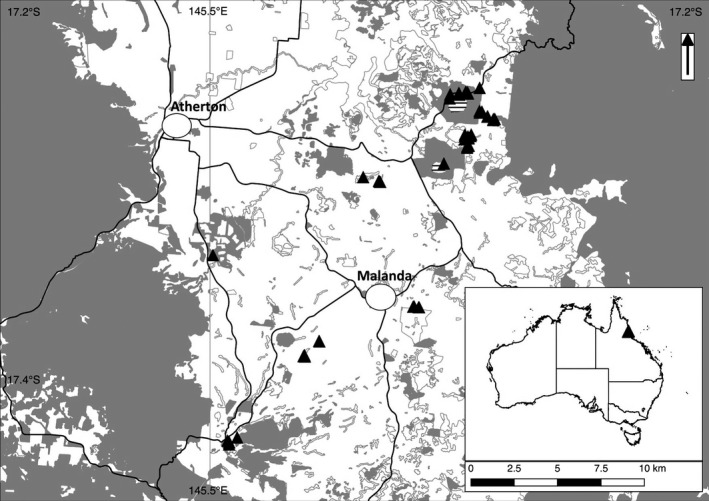
Locations of study sites on the Atherton Tablelands, Queensland, Australia. The gray polygons are remnant forest, the small white polygons with gray borders are nonremnant (mostly regrowth), the white background is cleared area (mostly pasture or grassland), the triangles are the study sites, hatched areas are open water lakes, and the black lines are main roads

Changes in ant community composition with age of restoration plantings were assessed across a chronosequence of 48 spatially‐discrete grassland, restoration (from 1 to 24 years of age), and remnant rainforest sites (Table 1), at 700–1,010 m elevation (Figure 1). Grassland sites were located in close proximity (20–200 m) to restoration sites, and restoration sites were between 25 m and 2.6 km from the nearest old‐growth forest. Old‐growth sample plots were placed at least 50 m in from the forest edge.

**Table 1:** Chronosequence class groupings and sampling periods of the sites

Class	Type/age (years) since planting	Median age (years)	No. sites – November only	No. sites –November + January	No. sites –January only	Total sites
0	Grassland	0	6	5	2	13
1	1–4	3	3	1	1	5
2	5–10	8	3	2	0	5
3	11–16	13	4	1	1	6
4	17–24	19	1	2	2	5
5	Rainforest	NA^a^	9	2	3	14

a Rainforest (old‐growth) sites were assigned an age of 50 years for quantitative analysis.

Restoration sites were within planned and managed ecological restoration plantings in grazed grassland that had a diversity of local tree species, similar tree spacings (~1.5–2 m) in the original plantings, few gaps from tree deaths, no weed infestations and were sufficiently large to fully contain a 15 m × 15 m ant sampling grid and allow a 10‐m buffer on all sides of the grid.

### 2.2 Site characterization

A 30 m transect that extended through the ant sampling grid was used to characterize the vegetation at each site. All woody stems with diameter at breast height (DBH) > 1 cm within 2.5 m of the transect were identified, DBH measured, and height estimated to the nearest meter. Canopy cover was measured every 2 m along the transect using a densiometer. Litter depth was measured every 2 m along the transect, and litter moisture content was graded on a scale from 1 (dry) to 6 (wet) using a visual examination and touch test. For all sites, the distance to the nearest remnant rainforest was calculated using desktop GIS software.

### 2.3 Ant sampling

Ants were sampled in 4‐week periods in the late‐dry season (November 2009) and the early wet season (January 2010). Thirteen sites across a range of ages (Table 1) were sampled in both periods to examine the possible influence of sampling period (i.e. season) on the ant communities. For both species (Appendix 1) and functional groups (Appendix 2), the effect of season was overwhelmed by differences among sites, and there was no consistent directional effect of season in ordination space on matched pairs of sites. In all subsequent analyzes, we ignored seasonal effects and, for sites sampled in both seasons, used only results from the first season.

Four sampling methods were employed to capture ants from a range of microhabitats, namely ground pitfall traps, baited arboreal pitfall traps, baited subterranean traps, and leaf litter extraction. A 4 × 4 grid of trapping points with 5 m spacing was established at each site. A ground pitfall trap (plastic container 45 mm in diameter and 55 mm deep, half‐filled with 50% ethylene glycol solution) was buried with its rim flush to the ground surface at each point. An inverted Petri dish was positioned above each pitfall trap to prevent rainfall from filling the traps but did not impede access by ants to the trap. An arboreal trap was taped to the nearest tree stem to each ground trap, at a height of 1.5 m. Arboreal traps were vials of 25 mm diameter and 50 mm depth, half‐filled with 50% ethylene glycol solution, and with a mixture of equal parts fish paste, peanut butter and honey around the inside rim. Arboreal trapping was not conducted in the youngest (<2 years) plantings, or in grassland, where there were no established trees. A subterranean trap was buried at 15 cm depth 1 m from each ground trap. Subterranean traps were baited Eppendorf tubes following Andersen and Brault (2010). Twelve 0.25 m^2^ leaf litter samples were collected on sunny days, in the immediate vicinity of, but not within, each trapping grid. Leaf litter was air‐dried, sieved, and placed in Winkler sacks for 48 hr. Litter sampling was not conducted at grassland sites as there was no distinct litter layer.

Ant specimens were identified to species level and voucher specimens lodged at the CSIRO Tropical Ecosystems Research Centre in Darwin. Many species could not be confidently named and were uniquely identified as morpho‐species. Ant species were classified into one of nine functional groups, following Andersen (1995): Dominant Dolichoderinae, Generalized Myrmicinae, Opportunists, Subordinate Camponotini, Hot‐, Cold‐ and Tropical‐Climate Specialists, Cryptic Species, and Specialist Predators.

### 2.4 Data analysis

We developed a Forest Development Index (FDI) using Principal Components Analysis of seven vegetation metrics: tree species richness, site basal area, mean height of vegetation, maximum height of vegetation, canopy cover, litter moisture, and mean litter depth. The first principal component (PC1) accounted for 79.5% of the variation in forest development among sites. All environmental variables were negatively correlated with PC1, and PC1 was significantly correlated with stand age (Appendix 3; *r*_*s*_ = −.945; *p* < .0001). The FDI was based on PC1 scores, adjusted by addition of a constant so that indices represented a sequence of vegetation development from 0 (grassland) to rainforest.

All ant analyzes were based on frequency of occurrence of ant species at sites, defined as the number of traps (*n* = 60) at a site in which a species was recorded. For grassland and young restoration sites, no adjustment was made for the lack of arboreal traps as this would have biased weightings by trap type; instead we assumed that no ants were caught in arboreal traps as there were no trees for such ants to inhabit. As a measure of functional group abundance, we summed the frequencies of occurrence of component species.

Variation in ant species and functional composition among sites was explored using nonmetric multidimensional scaling (NMDS) based on Bray–Curtis dissimilarity and performed in PRIMER 6 (Clarke & Gorley, 2006). We evaluated the relative importance of the FDI, elevation and distance from mature rainforest on species and functional composition using distance‐based linear models, implemented in the DISTLM module of the PERMANOVA+ add‐on to PRIMER 6 (Anderson, Gorley, & Clarke, 2008). We appraised all possible combinations of FDI, elevation and distance using the Akaike Information Criterion for small samples (AIC_C_) with 9,999 permutations. DISTLM results were visualized using distance‐based redundancy analysis (Anderson et al., 2008).

To identify indicator species, we used Indicator Species Analysis (McCune & Grace, 2002) to examine the affiliation of each ant species to either grassland or primary forest, based on frequency data. Twenty‐two species with a significant indicator value were selected for further analysis, comprising 14 species that were indicative of forest and eight species indicative of grassland habitat (Appendix 4).

The relationship between frequency of occurrence of each species at a site and a site's FDI was examined using logistic regression of binomial proportions of occurrence, with a logit link function. From this analysis, we developed a novel indicator species index that addresses the problems of using species‐level information for highly diverse taxa with high rates of species turnover and can be used to assess sites with varying sampling methodology. To make the Ant Forest Indicator Index (AFII) as robust as possible, we selected only those species that were strongly associated with either grassland or rainforest (i.e. with a >95% likelihood (*p* < .05) of being found in those habitats), and absent from the other. We calculated the AFII based on the presence of these species, defined as the number of forest species minus the number of grassland species at a site (see Appendix 5 for full details). We assessed the relationship between this index and FDI through ordinary least squares linear regression.

## 3 Results

**Figure 2:**
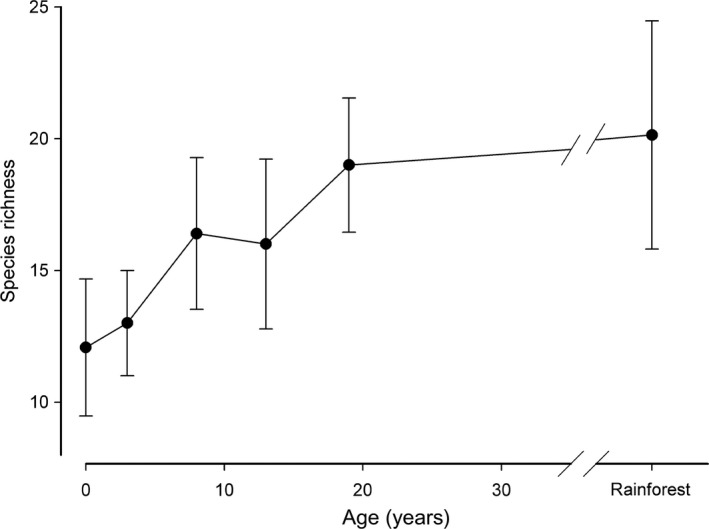
Site species richness (mean ± *SD*) of ants in age classes (from Table 1) along a chronosequence of regeneration from grassland to rainforest (*r*_s_ = .73, *n* = 48 *p* < .001)

In total, 109 ant species were recorded, with site richness ranging from 4 to 28. Species richness increased with age of regeneration to approach that of old‐growth rainforest sites by 17–24 years (Figure 2). All nine possible ant functional groups were detected, with a range from 1 to 27 species per group.

Two introduced ant species, *Pheidole megacephala* and *Tetramorium bicarinatum,* were abundant at some of the sites. *Tetramorium bicarinatum* was frequently recorded in grassland (10 of 13 sites), with three additional records in restoration plantings at relatively low abundance. In contrast, *P. megacephala* was recorded in four restoration plantings and one grassland site, and strongly dominated ant community composition at two of these restoration sites. At a 4‐year‐old restoration site, 97% (*n* = 1,692) of ant individuals were *P. megacephala*. At a 3‐year‐old site, *P. megacephala* was also the most abundant species, comprising 41% of individuals (*n* = 1,219). At both of these sites, 100% of subterranean captures were of *P. megacephala*.

**Figure 3:**
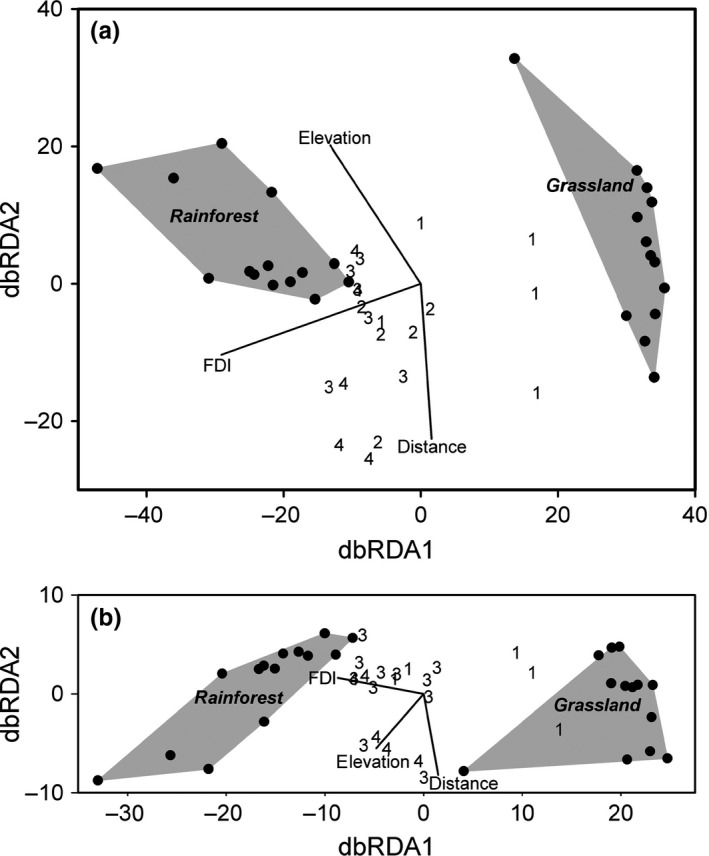
(a) Distance‐based Redundancy Analysis (dbRDA) for the full fitted model (from Appendix 4) for ant species composition. Rainforest and grassland sites are represented by closed circles linked by a shaded convex polygon. Regeneration sites are numbered 1–4, age classes corresponding to median ages of 3, 8, 13, and 19 years, respectively (Table 1). Vectors are for the model variables (FDI, Forest Development Index; distance, distance from rainforest) indicating alignment with dbRDA axes. dbRDA1 accounted for 74.4% of the fitted variation and was associated primarily with the Forest Development Index, while dbRDA2 accounted for a further 18.9% and was primarily associated with elevation and distance from rainforest. (b) Distance‐based Redundancy Analysis (dbRDA) for the full fitted model (from Table 2) for ant functional group composition. dbRDA1 accounted for 88.9% of the fitted variation and dbRDA2 a further 7.6%

From distance‐based redundancy analysis, it was clear that ant species composition in grassland was very different from that in rainforest, and species composition became increasingly forest‐like with increasing age of restoration (Figure 2). FDI was strongly associated with ant species and functional group composition along this successional pathway, with relatively weak associations with elevation and distance to old‐growth forest (Table 2). The effect of elevation was mostly within rather than between age classes of sites (Figure 3a). Regeneration sites older than 10 years were closer in composition to rainforest than to grassland (Figure 3a). Similar trends were evident for ant functional group composition (Table 2; Figure 3b).

**Table 2:** Distance‐based Linear Models of ant (a) species composition, and (b) functional group composition, ranked by AIC_C_. Parsimonious models (ΔAIC_C_ < 2.0; above gray dashed line) all included the Forest Development Index (FDI) but the most parsimonious model included only the FDI. %Dev is the % of deviance explained

(a) Species composition			(b) Functional groups		
Models	ΔAIC_C_	%Dev	Models	ΔAIC_C_	%Dev
FDI + distance	0	20.5	FDI + elevation	0	27.3
FDI	0.02	16.6	FDI	0.25	23.3
FDI + elevation	0.05	20.4	FDI + elevation + distance	0.99	29.4
FDI + elevation + distance	0.55	23.5	FDI + distance	1.02	25.7
Distance	5.79	6	Elevation + distance	8.8	12.6
Elevation	6.04	5.5	Distance	8.96	8.1
Elevation + distance	6.28	9.4	Elevation	9.4	7.2

**Figure 4:**
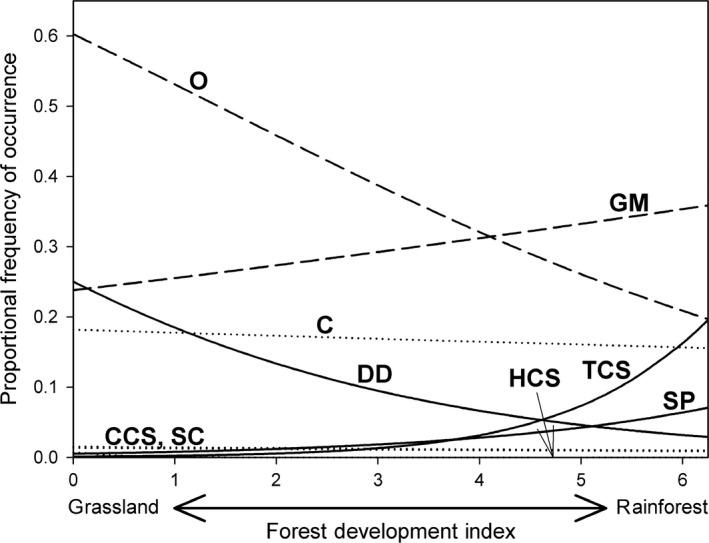
Modeled frequency of occurrence of ant functional groups based on binomial proportions regression (Table 3). Solid lines indicate groups with significant slopes and the absence (HCS) indicates low frequency of occurrence in either grassland or rainforest. Dashed lines indicate groups with significant slopes but abundant throughout the range of the Forest Development Index. Dotted lines indicate nonsignificant (*p *>* *.05) slopes. Functional group codes are given in Table 3

Five functional groups varied strongly and sequentially with regeneration development (Figure 4; Table 3). Of these, three were most informative for distinguishing grassland from forest: Specialized Predators and Tropical‐climate Specialists were rare or absent from grassland sites, and Dominant Dolichoderinae were rarely recorded in rainforest. Generalized Myrmicinae and Opportunists showed strong relationships with forest development but were abundant in both grassland and rainforest. The frequencies of occurrence of four groups – Cryptic Species, Cold‐climate Specialists, Hot‐climate Specialists, Subordinate Camponotini – were independent of forest development stage, and with the exception of Cryptic Species, were too rarely recorded for meaningful interpretation of analyzes (Figure 4).

**Table 3:** Fit of binomial regression slopes for frequency of occurrence of ant functional groups at the sample sites

Functional group	Estimate (slope)	SE of estimate	t	*p*
Cryptic species (C)	−0.030	0.029	0.30	.296
Cold‐climate specialists (CCS)	−0.071	0.094	−0.75	.452
Dominant dolichoderinae (DD)	−0.386	0.034	−11.24	**<.001**
Generalized myrmicinae (GM)	0.093	0.025	3.79	**<.001**
Hot‐climate specialists (HCS)	−2.7	6.160	−0.44	.661
Opportunists (O)	−0.292	0.023	−12.56	**<.001**
Subordinate camponotini (SC)	−0.073	0.100	−0.73	.463
Specialist predators (SP)	0.432	0.102	4.26	**<.001**
Tropical‐climate Specialists (TCS)	0.896	0.116	7.71	**<.001**

Bold values indicate a statistically significant fit.

**Figure 5:**
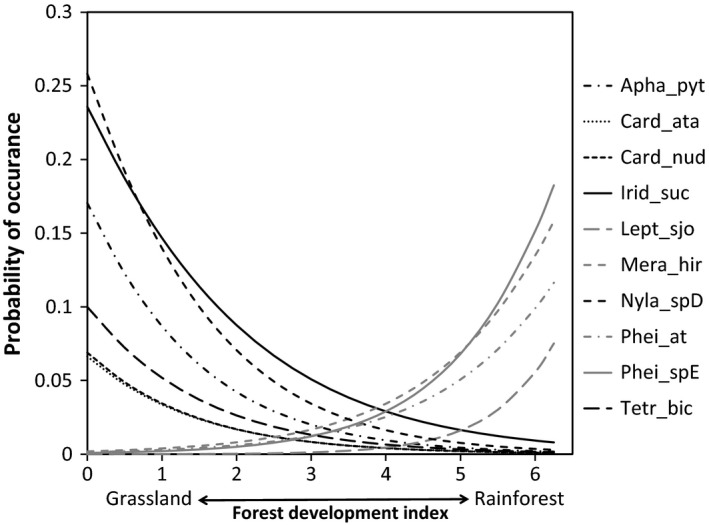
Modeled frequency of occurrence of ant species based on binomial proportions regression. Grassland associated species (black lines): Nyla_spD = *Nylanderia* sp. D, Irid_suc = *Iridomyrmex suchieri*, Apha_pyt = *Aphaenogaster pythia*, Card_nud = *Cardiocondyla nuda*, Card_ata = *Cardiocondyla atalanta,* Tetr_bic = *Tetramorium bicarinatum*. Forest associated species (gray lines): Phei_spE = *Pheidole* sp. E, Mera_hir = *Meranoplus hirsutus*, Phei_at = *Pheidole athertonensis,* and Lept_sjo = *Leptogenys sjostedti*

**Figure 6:**
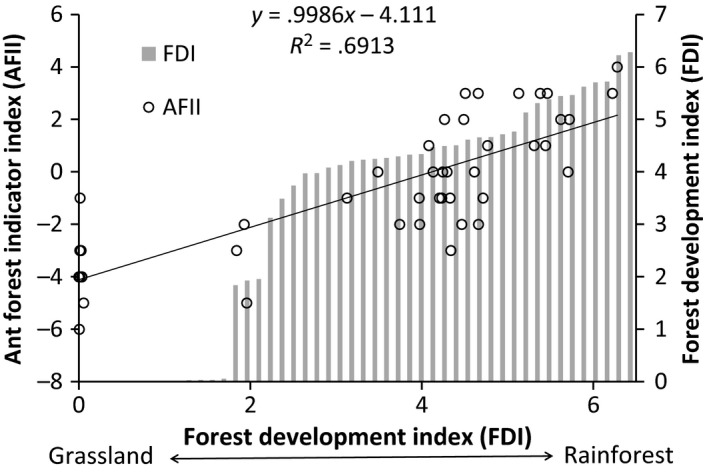
Ant Forest Indicator Index (AFII) showing the ant species assemblage response to changing forest development (FDI). The FDI is arranged along the *x*‐axis in order of increasing FDI from grassland sites with a FDI value approaching zero and old‐growth sites ~6

From logistic regressions of species occurrence, combined with expert opinion, we selected ten species (Appendices S6 and S7) from the 22 species identified as indicator species: six very strongly associated with grassland (Indicator Value for grassland > 35%, and for forest = 0%–1%; *Nylanderia* sp. D, *Iridomyrmex suchieri*,* Aphaenogaster pythia*,* Cardiocondyla nuda*,* Cardiocondyla atalanta,* and *Tetramorium bicarinatum*); and four strongly associated with rainforest (Indicator Value for forest > 50%, and for grassland = 0%; *Pheidole* sp. E, *Meranoplus hirsutus*,* Pheidole athertonensis,* and *Leptogenys sjostedti*; Figure 5). Using these ten species, we calculated the AFII for each site, which ranged from −6 (i.e. supporting all grassland and no forest species) at a grassland site, to 4 (i.e. supporting all forest and no grassland species) at a forest site. Overall, the AFII was highly and linearly correlated with the FDI (*R* ^2^ = .69, *n* = 48; Figure 6). This relationship had very high predictive power because AFII varied so systematically at regenerating sites, rather than just differentiating forest from grassland sites.

## 4 Discussion

Using a chronosequence approach, we demonstrate convergence of ant community composition at sites undergoing restoration from grassland toward a mature forest state. Our findings show that ecological restoration extends beyond the planted trees, and indicate a promising developmental trajectory toward broad ecological convergence with forest. We have used the data to develop a novel Ant Forest Indicator Index that has wide applicability for assessing forest restoration.

The seral development of ant assemblages described here accords with King et al. (1998), who also reported distinct grassland, restoration and rainforest ant communities in the study region. However, it contrasts with rapid assessments at the ant genus‐level, which were of limited use in discriminating among reforestation types (Nakamura et al., 2003; Piper et al., 2009). This contrast reinforces the value of species‐level information when assessing restoration success (Andersen, Hoffmann, Müller, & Griffiths, 2002; Andersen & Majer, 2004).

Experimental studies have demonstrated that shade is a primary requirement for the colonization of rainforest ants in forest restoration, and also that canopy cover suppresses the occurrence of pasture‐associated ant species (Nakamura, Catterall, Burwell, Kitching, & House, 2009; Nakamura, Catterall, House, Kitching, & Burwell, 2007). Forest restoration on the Atherton Tablelands has used a variety of methods and planting densities (Preece, Crowley, Lawes, & van Oosterzee, 2012). High‐density plantings of local forest species can achieve a closed canopy within 5 years and promote rainforest‐like conditions (Kanowski, 2010; Kanowski, Catterall, Wardell‐Johnson, Proctor, & Reis, 2003). In our study, ant communities in restoration stands that had a well‐developed closed canopy most closely approached those of old‐growth forests.

Despite substantial convergence, ant assemblages in restoration sites had not fully reached a mature forest state after 24 years (our oldest site). Restoration sites on the Atherton Tablelands would appear to require at least 50 years before they are potentially analogous with quality faunal habitat approaching old‐growth rainforest, and for some taxa this may require 100 or more years (Bowen, McAlpine, House, & Smith, 2007). Successful restoration of faunal assemblages is contingent upon dispersal as well as the development of suitable habitat, and dispersal limitation related to proximity to intact habitat has been implicated in the variable recovery of ant communities in mine site rehabilitation (Andersen et al., 2003). We found a relatively minor influence of distance from old‐growth forest on ant assemblage structure at restoration sites, which suggests a general lack of dispersal limitation. None of our sites were more than 2.6 km from mature forest. Such distances are evidently within the range of the winged queens that are typical of ants. However, some specialist rainforest ant taxa such as species of *Cerapachys* and *Pseudoneoponera* do not have winged queens (Peeters & Ito, 2001), and therefore would not be expected to colonize isolated restoration sites. The only restoration sites where we recorded such species were located immediately adjacent to mature forest, and their absence from other restoration sites may not necessarily reflect unsuitable habitat condition. Such dispersal limitation of habitat specialists underlies the need to design restoration programs to optimize forest connectivity (Brodie et al., 2015; Ikin et al., 2016).

Successful restoration of ant communities on the Atherton Tablelands is potentially also limited by the occurrence of the introduced *Pheidole megacephala*, which is widespread in the study region. This ant has the capacity to invade undisturbed rainforest and devastate the native ant fauna (Haskins & Haskins, 1965; Hoffmann, Andersen, & Hill, 1999; Hoffmann & Parr, 2008). The latter was observed at two 3–4 years old restoration sites, where *P. megacephala* comprised most of the recorded specimens and all of the subterranean ones. The genus *Pheidole* has been tentatively reported as a potential rainforest indicator (Piper et al., 2009). However, the species‐level responses of *Pheidole* spp. are far more informative than the genus‐wide response, because of the confounding influence of *P. megacephala* and that indigenous rainforest *Pheidole* species were recorded in all habitats. Indeed, six rainforest *Pheidole* species were included among the 22 indicator species (Appendix 4), and two of these (*Pheidole athertonensis, Pheidole* sp. E) were among the final ten selected indicator species (Appendix 6). As the introduced *P. megacephala* tends to be most abundant in early stage restoration sites, restoration methods that encourage the rapid development of a closed canopy and the early colonization of rainforest *Pheidole* species are recommended.

Ant functional group composition showed systematic variation along our chronosequence, and such variation was consistent with that predicted by the functional group model (Andersen, 1995). Opportunists typically respond positively to disturbance (Andersen, 1995) and were most abundant in pastures and young restoration sites. The highly thermophilic Dominant Dolichoderinae strongly prefers open, well‐insolated habitats; it was common in grassland but rapidly declined in abundance with forest regeneration. Conversely, Tropical‐Climate Specialists and Specialist Predators are known to be highly sensitive to disturbance (Andersen, 2000; Leal, Filgueiras, Gomes, Iannuzzi, & Andersen, 2012), and occurred almost exclusively in mature rainforest, where they were common. Similar responses to forest restoration have been described for these functional groups in previous studies in the region (Andersen, 1995; King et al., 1998; Piper et al., 2009).

An objective of our study was to develop a method for using species‐level information for bioindication when species are highly diverse and patchily distributed and that has high predictive power for use in other studies in the region. Our novel composite Ant Forest Indicator Index has four important advantages over using individual species indicators: (1) It uses multiple species, each of which may be patchily distributed in their favoured habitat and relative to the FDI, but collectively they occur at all sites; (2) the derived indicator score varies linearly with forest development, and so can be readily associated with successional development; (3) because the indicator score is a relative one (i.e. number of forest taxa relative to the number of grassland taxa), it can be applied to any assessment of successional status of rainforest restoration sites in the study region, to a large degree independently of variation in sampling methodology and intensity, so long as there is a relatively robust representation of species composition; and (4) the AFII can be used for any region where ant occurrence data are available for forest and matrix habitats, thus allowing the selection of its component indicator species. A key advantage of the AFII is that it can be used to assess the status of any site where ants have been sampled in the region, without the need of a full chronosequence study, or any new comparative data from reference sites. As a test of the efficacy of our particular AFII for evaluating the success of other restoration programs on the Atherton Tablelands, we applied it to the data from King et al. (1998). That study recorded only five of the 10 species that we used to derive our index, but still produced consistent results: the pasture site scored −2 (it supported two of our grassland species, and none of our forest species), the two sites undergoing revegetation (both ≤1 year old) both scored −1 (each with one of our grassland species, and none of our forest species), and the two rainforest sites both scored +1 (each with one of our forest species and none of our grassland species). We believe that our index has wide applicability for the incorporation of species‐level information from highly diverse and patchily distributed species in reforestation plantings of various ages, wherever data are available on occurrences of local species in forest and matrix habitats.

While ant species and functional groups have been successfully used here as ecological indicators of rainforest restoration in the Wet Tropics, restoration success must be considered in the context of the range of ecological functions to which such indicators apply (Holt & Miller, 2010; Lindenmayer & Likens, 2011), and we acknowledge that restoration of the full suite of ecosystem functions relies on more than just ants. The fundamental importance of ants to ecosystem function (Del Toro, Ribbons, & Pelini, 2012; Folgarait, 1998) makes them a highly suitable indicator taxon, but other faunal taxa may respond to forest restoration differently (Freeman, Catterall, & Freebody, 2015; Laurance, 1994; Whitehead et al., 2014). Although our Ant Forest Indicator Index is a highly useful tool for measuring the progress of forest restoration, further studies are required to document how reliably changes in ant communities reflect those of other important faunal groups.

## Conflict of Interest

None declared.

## Appendix 1

### 1.1

Nonmetric multidimensional scaling ordination in three dimensions (stress = 0.16) of sites on ant species frequency of occurrence. Arrows connect samples from the same sites in different seasons (Closed circles: Nov, Open circles: Jan) and their length indicates the relative difference between the samples.

## Appendix 2

### 2.1

Nonmetric multidimensional scaling ordination in two dimensions (stress = 0.16) of sites on ant functional groups frequency of occurrence. Arrows connect samples from the same sites in different seasons (Closed circles: Nov, Open circles: Jan) and their length indicates the relative difference between the samples.

## Appendix 3

### 3.1

The relationship between the Forest Development Index (based on the first axis scores of PCA of a range of habitat variables) and vegetation age for 48 sites from which ants were sampled on the Atherton Tableland, far north Queensland, Australia.

## Appendix 4 Indicator values for the 22 significant species identified by Indicator Species Analysis

### 4.1

**Table:**

Species	%IV grassland	%IV rainforest	Probability	Forest/Grassland
Anon_spA	0	50	.005	F
Apha_pyt	88	1	.001	G
Both_spA	0	43	.016	F
Card_ata	38	0	.017	G
Card_nud	38	0	.019	G
Hypo_spA	0	86	.001	F
Irid_suc	74	0	.001	G
Lept_sjo	0	50	.005	F
Mera_hir	0	50	.007	F
Nyla_spD	67	1	.001	G
Orec_rob	0	36	.032	F
Phei_at	0	67	.002	F
Phei_spA	5	50	.025	F
Phei_spD	1	60	.006	F
Phei_spE	0	79	.001	F
Phei_spI	38	0	.017	G
Phei_spM	0	62	.002	F
Rhyt_pup	0	64	.001	F
Sole_spA	65	13	.017	G
Sole_spB	0	50	.005	F
Stru_spB	1	53	.008	F
Tetr_bic	69	0	.001	G

## Appendix 5 A guide to calculating the Ant Forest Indicator Index (AFII)

### 5.1

1. Use Indicator Species analysis (McCune & Grace, 2002) to identify grassland (or matrix) and forest ant species. The same can be done for functional groups.
2. We recommend using a threshold indicator value of >50% at *p* < .05 to select indicative species in the first instance. Specialist knowledge about habitat affiliations can also be used to select additional species that approach but don't quite meet the threshold indicator value.
3. To make the index as robust as possible, using the indicator values (IV) and specialist knowledge, select the species mostly strongly associated with each habitat – these are those species only found in one habitat and not in the other – see species shaded in grey in the table below (e.g. for strongly forest associated species IV_forest_ > 50% and IV_grass_ ~ 0%; for strongly grass associated species IV_forest_ = 0% and IV_grass_ ≥ 38%).
  **Table:**
  Species	%IV grassland	%IV rainforest	Probability	Forest/Grassland
  Anon_spA	0	50	.005	F
  Apha_pyt	88	1	.001	G
  Both_spA	0	43	.016	F
  Card_ata	38	0	.017	G
  Card_nud	38	0	.019	G
  Hypo_spA	0	86	.001	F
  Irid_suc	74	0	.001	G
  Lept_sjo	0	50	.005	F
  Mera_hir	0	50	.007	F
  Nyla_spD	67	1	.001	G
  Orec_rob	0	36	.032	F
  Phei__at	0	67	.002	F
  Phei_spA	5	50	.025	F
  Phei_spD	1	60	.006	F
  Phei_spE	0	79	.001	F
  Phei_spI	38	0	.017	G
  Phei_spM	0	62	.002	F
  Rhyt_pup	0	64	.001	F
  Sole_spA	65	13	.017	G
  Sole_spB	0	50	.005	F
  Stru_spB	1	53	.008	F
  Tetr_bic	69	0	.001	G
4. For each site, count the number of strongly grassland and forest associated species separately. Arrange these counts in a table by site.
5. Calculate the Ant Forest Indicator Index (AFII) by site by subtracting the number of grass species (Grass_Spp.) at a site from the number of forest species (Forest_Spp.) at that site. Negative values for the AFII indicate sites dominated by grass species, positive values indicate sites dominated by forest species, and zero indicates neither grass species or forest species dominance and is typically associated with the grassland‐forest ecotone.

**Table:**

Site	Grass_Spp.	Forest_Spp.	AFII
Thur‐G2	4	0	−4
D‐G1	4	0	−4
C‐G1	6	0	−6
LB‐G2	4	0	−4
D‐G2	4	0	−4
C‐G2	4	1	−3
C‐G3	4	0	−4
Ch‐G1	1	0	−1
Ch‐G2	3	0	−3
B‐G1	3	0	−3
T‐G1	4	0	−4
Thur‐G1	4	0	−4
LB‐G1	5	0	−5
D‐08	3	0	−3
C‐08	3	1	−2
B‐07	5	0	−5
LB‐05	1	0	−1
C‐99	1	1	0
C‐03	2	0	−2
Ch‐00	1	0	−1
B‐98	2	0	−2
LB‐93	2	3	1
C‐06	1	1	0
C‐96	1	0	−1
C‐02	2	1	−1
C‐05	1	1	0
LB‐94	0	2	2
LB‐88	0	0	0
Thur‐86	1	0	−1
D‐98	3	0	−3
Ch‐91	2	0	−2
T‐RF3	0	2	2
LE‐RF1	0	3	3
Ch‐96	0	0	0
LB‐RF2	0	3	3
D‐95	3	1	−2
B‐RF1	1	0	−1
Thur‐92	0	1	1
C‐RF3	1	4	3
D‐RF1	1	2	1
LB‐RF1	0	3	3
D‐RF3	1	2	1
T‐RF1	0	3	3
D‐RF2	0	2	2
C‐RF2	2	2	0
C‐RF1	0	2	2
T‐RF2	0	3	3
W‐RF1	0	4	4

## Appendix 6 Fit of binomial regression slopes for frequency of occurrence of the 10 species selected as indicator species

### 6.1

**Table:**

Species	Estimate (slope)	*SE* of estimate	t	*p*	*R* ^2^
Constant	−5.998	0.675	−8.89	**<.001**	
*Aphaenogaster pythia*	4.416	0.687	6.43	**<.001**	.88
*Cardiocondyla atalanta*	3.352	0.702	4.77	**<.001**	.84
*Cardiocondyla* nuda	3.395	0.701	4.84	**<.001**	.84
*Iridomyrmex suchieri*	4.822	0.684	7.05	**<.001**	.89
*Leptogenys sjostedti*	−4.5	1.48	−3.05	**.002**	.18
*Meranoplus hirsutus*	−0.308	0.851	−0.36	.717	.57
*Nylanderia* sp. D	4.942	0.684	7.23	**<.001**	.89
*Pheidole athertonensis*	−0.534	0.897	−0.6	.551	.51
*Pheidole* sp. E	−1.084	0.889	−1.22	.223	.52
*Tetramorium bicarinatum*	3.801	0.694	5.48	**<.001**	.86

Bold values indicate a statistically significant fit.

## Appendix 7

### 7.1

Plots of model fit based on binomial proportions regression for the 10 species selected as indicator species
